# Anti-Fatigue Effects of the Unique Polysaccharide Marker of *Dendrobium officinale* on BALB/c Mice

**DOI:** 10.3390/molecules22010155

**Published:** 2017-01-18

**Authors:** Wei Wei, Zhi-Peng Li, Tong Zhu, Hau-Yee Fung, Tin-Long Wong, Xin Wen, Dik-Lung Ma, Chung-Hang Leung, Quan-Bin Han

**Affiliations:** 1 School of Chinese Medicine, Hong Kong Baptist University, 7 Baptist University Road, Kowloon Tong, Hong Kong, China; xyzdf23@163.com (W.W.); lizhipeng0903@163.com (Z.-P.L.); Oliver9023@gmail.com (T.Z.); 15485862@life.hkbu.edu.hk (H.-Y.F.); 15485021@life.hkbu.edu.hk (T.-L.W.); 16483634@life.hkbu.edu.hk (X.W.); 2 Department of Chemistry, Hong Kong Baptist University, Kowloon Tong, Hong Kong, China; edmondma@hkbu.edu.hk; 3 State Key Laboratory of Quality Research in Chinese Medicine, Institute of Chinese Medical Sciences, University of Macau, Taipa, Macau, China; duncanleung@umac.mo

**Keywords:** *Dendrobium officinale*, authentication marker, polysaccharide, DOP, anti-fatigue activity

## Abstract

*Dendrobium officinale* extract shows potent anti-fatigue effects; however, the active substance responsible for these effects remains undetermined. A glucomannan with a huge molecular size of 730 kDa, called DOP, was identified as the unique authentication marker of this expensive herb. DOP exhibited immunomodulating effects on macrophages and lymphocytes in our previous study. Clinical reports also showed that people with fatigue syndrome have a disturbed immune system. Because DOP is the unique and dominant component of *D. officinale*, we hypothesize that DOP may also have anti-fatigue activity. The present study aims to evaluate the anti-fatigue activity of DOP on BALB/c mice, with *Rhodiola rosea* extract as a positive control. DOP and *Rhodiola rosea* extract were orally administered at doses of 50 mg/kg and 100 mg/kg, respectively, for four weeks, and the anti-fatigue activity of DOP on BALB/c mice was evaluated using the weight-loaded swimming test. The contents of lactic dehydrogenase (LDH), creatine phosphokinase (CK), triglyceride (TG), blood urea nitrogen (BUN), superoxide dismutase (SOD), malondialdehyde (MDA), lactic acid (LD), and glutathione peroxidase (GSH-Px) in serum, glycogen of liver and gastrocnemius muscle were also determined. Their effects on variability of T cells and B cells were determined by using tetrazolium compound (MTS) method. The weight-loaded swimming exercise caused fatigue syndrome, mainly including the decreases of serum SOD/GSH-Px and gastrocnemius glycogen, as well as the increases of LDH, BUN, MDA, CK, TG, and LD in serum. All of these indicators of fatigue were inhibited to a certain extent by both DOP and *Rhodiola rosea* extract; however, the effects of DOP were much stronger than those of *Rhodiola rosea* extract. Compared to the positive control, mice dosed with DOP showed increases in endurance, body weight, and food intake. Furthermore, DOP-feeding mice significantly increased the cell variability of T lymphocytes and B lymphocytes, compared with that of mice in control group. This study indicates that the unique and dominant polysaccharide DOP of *D. officinale* has stronger anti-fatigue activity than *Rhodiola rosea* extract. As such, DOP has promising potential for pharmaceutical development into health products to reduce fatigue.

## 1. Introduction

Fatigue syndrome refers to difficulty in initiating or sustaining voluntary activities [1]. It is a multifaceted illness because its pathophysiology and etiology are still unclear, accompanying many diseases, such as aging, advanced cancer, depression, AIDS, multiple sclerosis, heart disease, diabetes, and Parkinson’s disease. In one study, more than 24% of patients in primary-care clinics indicated that fatigue is a major problem [2]. Fatigue syndrome is a serious worldwide prevalent health problem affecting over 800,000 American people and approximately 240,000 patients in the UK; 85%–90% of these people are not receiving medical care that effectively reduces fatigue [3].

Another source of fatigue in modern populations is exercise. More and more people now exercise regularly to enhance their health. Excessive exercise also causes fatigue and even various types of damage to the body. Therefore, in the past few decades, health scholars and athletic physiologists have been looking for natural active compounds that can improve athletic ability, postpone fatigue, and accelerate the body’s recovery from physical exertion. However, many of the active substances reported to address fatigue have side effects. For instance, *Rhodiola* L. extract of which salidroside is the main functional component, showed anti-fatigue effects [4,5], but excessive *Rhodiola* L. may result in hypoglycemia, which compromises recovery from fatigue. Therefore, safe and effective anti-fatigue natural products are still desired.

Various studies have sought evidence that people with fatigue syndrome have a disturbed immune system, such as dysfunction of natural killer cell activity [6,7], redundant cytokines production in vivo [8], and reduced mitogenic response of lymphocytes [9]. Moreover, the clinical treatment with cytokines, such as interleukin-1, interleukin-2, and interferon-γ gave rise to fatigue symptoms [10]. Therefore, the natural products with immunoregulation effects may have fatigue-resistance activity.

*Tiepi Fengdou*, the stem of *Dendrobium officinale*, has been used for thousands of years as a health tea herb in East Asia. It ranks as the first of “nine kinds of Chinese medicinal herbs” and is traditionally recorded as a tonic to nourish Yin, supply body fluids, strengthen immunity, and benefit gastric tonicity in traditional Chinese medicine theory [11]. Its water extract has been reported to exhibit anti-fatigue effects on mice [12], however, its anti-fatigue ingredients remain undetermined.

In our previous studies, a unique polysaccharide marker (glucomannan, called DOP) was identified for the authentication of *D. officinale* [13]. DOP is likely also to be the main active ingredient because its content exceeds 30% of the dry herb by weight, and it shows immunomodulating effects towards immune cells [14,15,16,17]. Therefore, we hypothesize that DOP also has anti-fatigue activity. In the present study, the anti-fatigue activity of DOP was evaluated using BALB/c mice in a repeated weight-loaded endurance swimming test. The contents of lactic dehydrogenase (LDH), creatine phosphokinase (CK), triglyceride (TG), blood urea nitrogen (BUN), superoxide dismutase (SOD), malondialdehyde (MDA), lactic acid (LD), and glutathione peroxidase (GSH-Px) in serum, the glycogen content of liver and gastrocnemius muscle, and the cell variability of T lymphocytes and B lymphocytes were also determined to clarify the underlying mechanism of action.

## 2. Results

### 2.1. Effects of DOP and Rhodiola Extract on Weight-Loaded Forced Swimming Endurance Time

**Figure 1:**
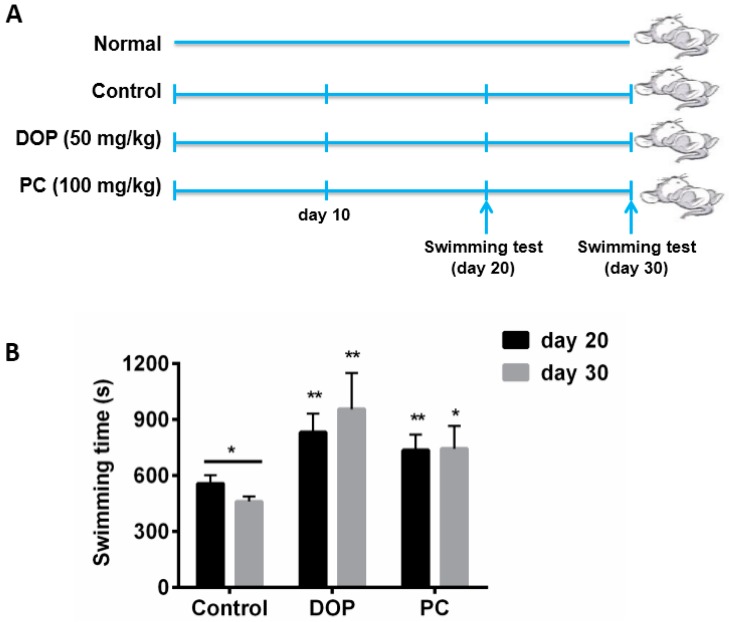
(**A**) Animal experimental design; (**B**) Effects of DOP and *Rhodiola* extract in weight-loaded swimming endurance time. Values are expressed as the mean ± SD. Normal group means that mice were unexposed to weight-loaded swimming endurance test. Control means that mice were given distilled water for 30 days. DOP means that mice were treated with DOP (50 mg/kg) for 30 days. PC means positive control, and mice of this group were treated with *Rhodiola* extract (100 mg/kg) for 30 days. Control group, DOP group, and PC group mice were all exposed to weight-loaded swimming test. * *p* < 0.05, ** *p* < 0.01, compared with the control group.

The weight-loaded forced swimming endurance test, one of the commonly-used anti-fatigue test models, was chosen to evaluate the anti-fatigue effect of DOP [18,19]. The duration of mean exhausting swimming test indicated the degree of anti-fatigue. As expected, the positive control (PC) *Rhodiola* extract significantly increased the swimming time (736.5 ± 81.08 s, *p* < 0.01) in comparison with control group (557 ± 45.42 s) at day 20 (Figure 1). The DOP group exhibited an even longer swimming time at 832.33 ± 51.23 s. It is suggested that DOP’s anti-fatigue effect is stronger than the positive control. Different from the normal anti-fatigue test, these three groups continued to be fed for another 10 days. At day 30, the second swimming test was performed. The control group obviously did not recover from fatigue because the swimming time significantly decreased to (461.33 ± 22.23 s, *p* < 0.05), compared to the first time. The PC group did not show such a decrease and remained at the same level. Instead, strikingly, the swimming time of DOP group continued to increase to around 956.75 s. These results suggest that DOP is a better anti-fatigue substance than *Rhodiola* extract.

### 2.2. Effects of DOP and Rhodiola Extract on Body Weight and Organ Indexes

**Figure 2:**
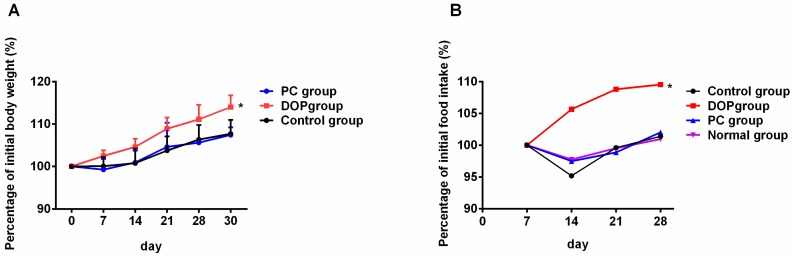
Effects of DOP and *Rhodiola* extract on percentage of initial body weight (**A**) and initial food intake (**B**) of BALB/c mice. Values in Figure 2A are expressed as the mean ± SD. Control means that mice were given distilled water for 30 days. DOP means that mice were treated with DOP (50 mg/kg) for 30 days. PC means positive control, and mice of this group were treated with *Rhodiola* extract (100 mg/kg) for 30 days. Control group, DOP group, and PC group mice were all exposed to weight-loaded swimming test. * *p* < 0.05 compared with the control group.

As shown in Figure 2A and Table S1, compared to body weights on the first day, the body weight of control group, DOP group, and *Rhodiola* extract group all increased during the experiment. When compared to the control group and PC group, DOP had an obvious effect on body weight gain (*p* < 0.05). Consistently, the food consumption rate of the DOP group increased during the course of the experiment when compared to the food consumption rate of control group, positive group, and normal group (Figure 2B and Table S2, *p* < 0.05).

**Figure 3:**
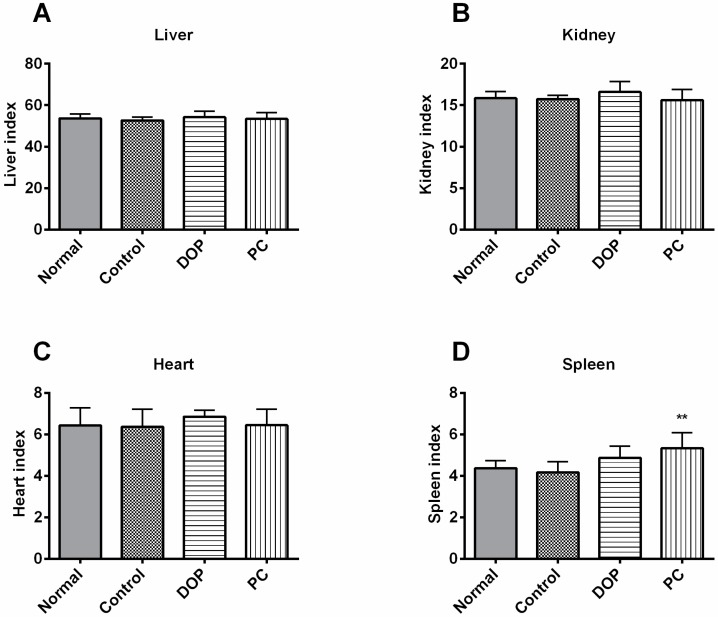
Effects of DOP and *Rhodiola* extract on liver index (**A**), kidney index (**B**), heart index (**C**), and spleen index (**D**) of BALB/c mice. Values are expressed as the mean ± SD. Control means that mice were given distilled water for 30 days. DOP means that mice were treated with DOP (50 mg/kg) for 30 days. PC means positive control, and mice of this group were treated with *Rhodiola* extract (100 mg/kg) for 30 days. Control group, DOP group, and PC group mice were all exposed to weight-loaded swimming test. ** *p* < 0.01 compared with the control group. Each organ index = weight of organ (mg)/body weight (g).

The organ indexes of liver, heart, kidney, and spleen were further evaluated. Oral administration of DOP and *Rhodiola* extract at 50 mg/kg and 100 mg/kg, respectively, for four weeks and swimming test slightly, but not significantly, ameliorated the organ index of heart (Figure 3C and Table S3), liver (Figure 3A and Table S3), kidney (Figure 3B and Table S3). As shown in Figure 3D and Table S3, the spleen index of the control group decreased slightly after weight-loaded swimming test compared to that of the normal group, but the spleen index of the DOP group was increased slightly compared to that of the control group. Strikingly, *Rhodiola* extract remarkably increased the organ index of spleen in comparison with that of control group (Figure 3D and Table S3, *p* < 0.01).

### 2.3. Effects of DOP and Rhodiola Extract on Serum Biochemical Parameters

**Figure 4:**
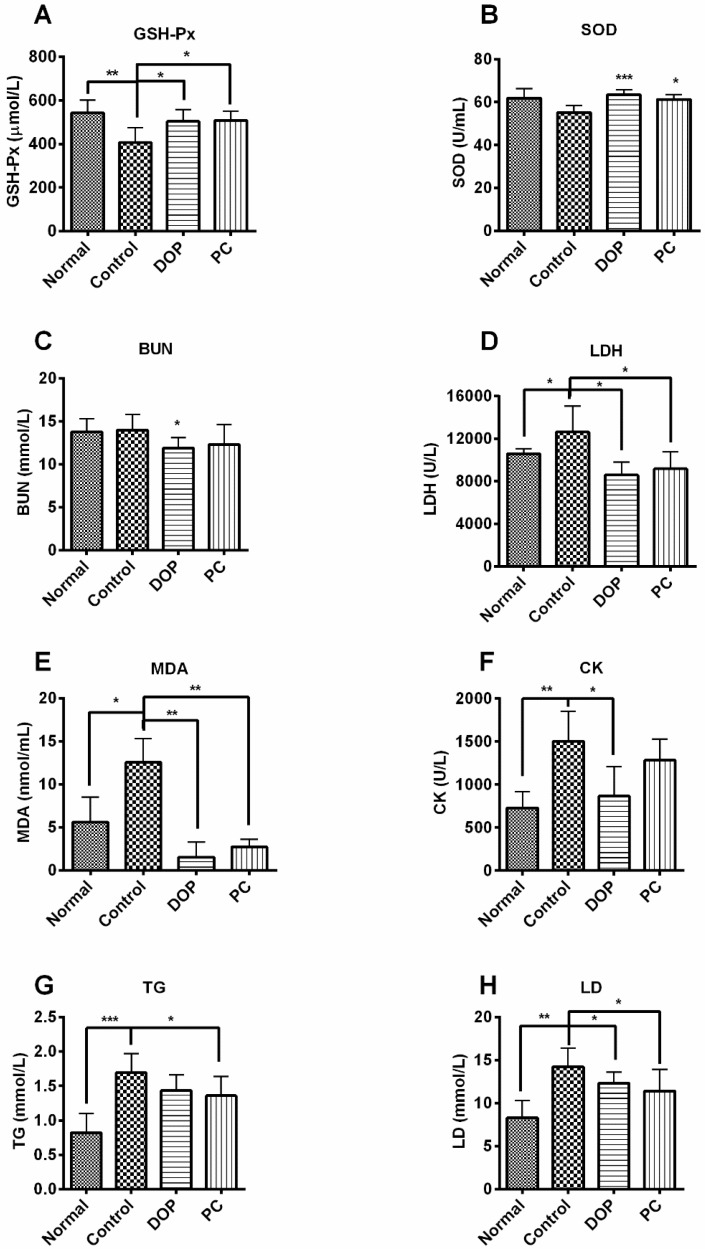
Effects of DOP and *Rhodiola* extract on serum biochemical parameters after weight-loaded swimming test. Values are expressed as the mean ± SD (*n* = 8). * *p* < 0.05, ** *p* < 0.01, *** *p* < 0.001, compared with the control group. GSH-Px: glutathione peroxidase (**A**) SOD: superoxide dismutase (**B**) BUN: blood urea nitrogen; (**C**) LDH: lactic dehydrogenase; (**D**) MDA: malondialdehyde; (**E**) CK: creatine phosphokinase; (**F**) TG: triglyceride; (**G**) LD: lactic acid; (**H**) Normal group means mice unexposed to the weight-loaded swimming test. Control group means mice exposed to the weight-loaded swimming test and treated with distilled water. PC means positive control, and mice of this group were treated with *Rhodiola* extract.

Blood biochemical parameters were determined to clarify the anti-fatigue mechanism. As shown in Figure 4, the weight-loaded and forced swimming test induced an increase of LDH, CK, TG, MDA, and LD levels in serum of mice in the control group, compared to the normal group. These effects were partially attenuated by DOP and *Rhodiola* extract. By contrast, exposure to the forced swimming test led to a decrease in GSH-Px level of the control group, and this effect was blocked by DOP and *Rhodiola* extract. Similar effects also observed in the SOD level, in which the decrease in control group was not so significant.

### 2.4. Effects of DOP and Rhodiola Extract on Glycogen in Liver and Gastrocnemius Muscle

**Figure 5:**
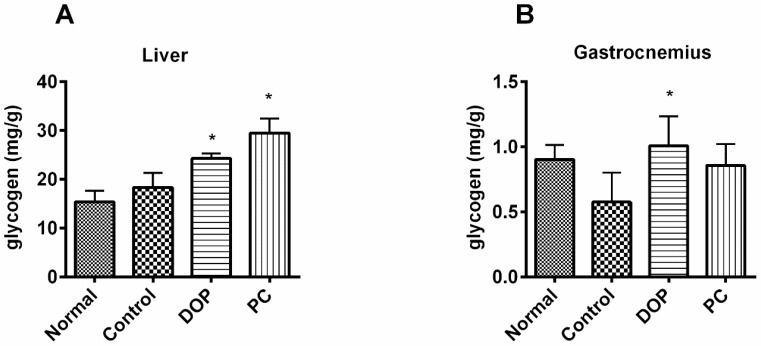
Effects of DOP and *Rhodiola* extract on liver glycogen (**A**) and gastrocnemius glycogen (**B**) after weight-loaded swimming test. Values are expressed as the mean ± SD (*n* = 8). * *p* < 0.05, compared with the control group. Normal group means mice unexposed to the weight-loaded swimming test. Control group means mice exposed to the weight-loaded swimming test and treated with distilled water. PC means positive control, and mice of this group were treated with *Rhodiola* extract.

Glycogen in liver and gastrocnemius muscle were determined by hepatic glycogen/muscle glycogen assay kits. As shown in Figure 5, the storage of hepatic glycogen increased after the swimming test. Simultaneously, DOP and *Rhodiola* extract enhanced the hepatic glycogen level in mice significantly compared to that of control group (Figure 5A, *p* < 0.05). DOP also boosted glycogen in the gastrocnemius muscle of mice compared to that of control group (Figure 5B, *p* < 0.05). In contrast, *Rhodiola* extract did not significantly increase the glycogen in the gastrocnemius muscle of mice in the study.

### 2.5. DOP’s Effect on Proliferation of Mouse Lymphocytes

**Figure 6:**
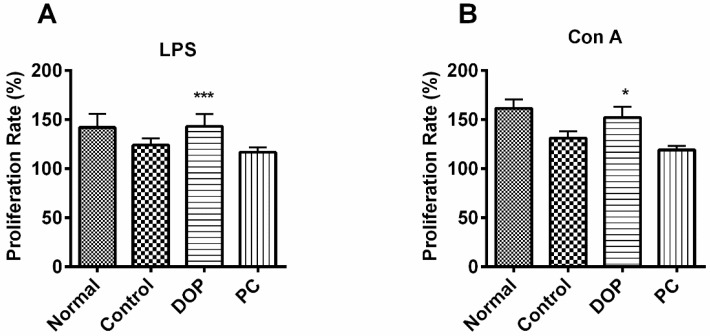
Proliferation of lymphocytes of mice fed with DOP and *Rhodiola rosea* extract after stimulation by LPS (**A**) or Con A (**B**). Lymphocytes (5 × 10^5^ cells/well) in 96-well-plate were incubated with LPS (20 μg/mL) and Con A (2.5 μg/mL) for 48 h. LPS, a lipopolysaccharide which is a mitogen of B cells, and Con A, a phytohemagglutinin which is a mitogen of T cells. The cell viability was measured with tetrazolium compound (MTS) method. The data are presented as the mean ± SD. * *p* < 0.05, *** *p* < 0.001, compared with the control group. PC means positive control.

After feeding mice with DOP and *Rhodiola* extract for 30 days, the lymphocytes from spleens of each group were subjected to a lymphocyte proliferation assay to assess the physical immunity. As shown in Figure 6, a significant increase of proliferation rates of lymphocytes stimulated by LPS and Con A was observed in DOP groups (*p* < 0.05), but it was not detected in the positive control group (*Rhodiola* extract), compared to the control group.

## 3. Discussion

The present study evaluated the anti-fatigue effects and underlying mechanism of DOP and *Rhodiola* extract in mice. DOP and *Rhodiola* extract extend the weight-loaded swimming time and facilitate oxidative enzyme activity and storage of hepatic glycogen. In addition, mice in the DOP group had a higher proliferation rate of T cells and B cells to mitogens, suggesting that both DOP and *Rhodiola* extract contribute to enhancement of physical strength and endurance.

Many Chinese research groups have demonstrated that *D. officinale*, *D. officinale* health tea, and compounds containing *D. officinale* have anti-fatigue and immunomodulating effects [12,20,21,22]. However, they did not find which phytochemical component of *D. officinale* is responsible for this anti-fatigue activity. The results of this study, in particular the increased swimming time, demonstrate that DOP treatment enhanced fatigue-resistance. This swimming test is a reliable measure of anti-fatigue treatment as established in both laboratory animals and humans [23,24,25]. In accordance with the previous study [4,5], the present study also showed that the positive control *Rhodiola* L. has anti-fatigue effects.

Stress represents the reaction of the body to stimuli that disturb its normal physiological equilibrium or homeostasis, often with detrimental effects. The weight of spleen, thymus, and thyroid of the immune system are decreased by immobilized stress [26]. Results in this study showed that the spleen index in the control group slightly decreased after the weight-loaded swimming test. *Rhodiola* extract increased spleen index significantly; this result was consistent with the previous study showing that *Rhodiola* enhanced cellular immunity in mice and rats [27,28,29]. However, for the lymphocyte proliferation assay, *Rhodiola* extract did not significantly increase proliferation rates of T cells and B cells compared with those of the control group. There is a study showing that a higher dose (200 μg) of *Rhodiola* extract was ineffective to enhance chemokinetic activity of spleen lymphocytes in mice [30]. In this study, 100 mg/kg (equals to 2000 μg) of *Rhodiola* extract was used as the positive control. Thus, too high a dosage may be the reason for the lack of response of B cells and T cells of mice in the *Rhodiola* extract group to mitogens in this study.

Fatigue syndrome is a worldwide problem, with a prevalence rate of 0.4%–1%. More than 70 million people worldwide are affected by fatigue. No physical examination signs are specific to fatigue and no diagnostic tests identify this syndrome. The pathophysiological mechanism of fatigue is also unclear.

Our results suggest that the mechanism of DOP against fatigue probably included three aspects. One possible explanation for the anti-fatigue effects of DOP and *Rhodiola* is that DOP and *Rhodiola* extract enhance triglyceride (TG) (or fat) mobilization during exercise, as indicated by the decrease in TG. Energy for muscular exercise is derived initially from the breakdown of muscle glycogen, and later from circulating glucose released by the liver and from non-esterified fatty acids [31]. After triglyceride mobilization, the utilization of hepatic glycogen and protein would be decreased. Thus, BUN levels in serum would be decreased. Simultaneously, glucose (Glc) storage would be increased in liver and gastrocnemius muscle. As is commonly known, glucose levels decreased immediately after exercise, and then, non-esterified fatty acids released for inhibiting reduction of circulating glucose. Such an effect that DOP and *Rhodiola* extract increased TG mobilization might become advantageous during prolonged exercise, since better utilization of TG saves consumption of glycogen and protein, and therefore delays fatigue.

The other possible explanation for the anti-fatigue effect of DOP is that it modifies several enzymes thereby preventing lipid oxidation which protects corpuscular membranes. Fatigue results in the release of reactive oxygen species (ROS) which cause lipid peroxidation of membrane structure. In fatigue conditions, MDA level is increased and is accompanied by a decrease in levels of the antioxidant enzymes GSH-Px and even SOD (with only an insignificant decrease trend). These conditions are also marked by the release of LDH and CK into the serum, serving as an indirect index of damage to membranes. Some reports showed that various antioxidant drugs—such as carvedilol, melatonin, and quercetin—have anti-fatigue effects because of their antioxidant activity [32]. After intake of DOP and *Rhodiola* extract, MDA, CK, and LDH levels were decreased and SOD and GSH-Px levels were increased thereby protecting the membrane structure and resisting oxidative damage.

The third reason for fatigue relief effect of DOP is that it has an immunomodulating effect. Although DOP did not increase the spleen index significantly, it significantly enhanced responses of B cells and T cells to mitogens in vivo compared with those of control group. The immunomodulating effects of DOP may be one of the key reasons it relieves fatigue. Various studies have sought evidence that people with fatigue syndrome have a disturbed immune system. Alteration of diverse immunological indicators, such as cytokine profile, function of natural killer cells, and responses of T cells to mitogens, has been reported [33]. The predominant pharmacological effect of glucomannan in *D. officinale* is the ability to modulate immune function [11,14,15,16,34]. Many polysaccharides have been reported to be able to activate macrophages and induce proliferation of lymphocytes, and this activation plays an important role in the immune response. In this study, mice of the control group showed an association between physical lassitude and immunity suppression. In addition, supplementation with DOP led to recovery of the reduced lymphocyte proliferation of chronic fatigue-challenged mice. The reason for the anti-fatigue effects of DOP may be its strong immunomodulatory effect in vivo.

The increase in body weight and food intake may explain the slight increase of the spleen index which may be relevant to the anti-fatigue effects of DOP observed in this study. Some reports showed that patients with fatigue syndrome have a disturbed immune system. A slight decrease of spleen index was also observed in the control group, which is associated with the fatigue caused by the swimming test. After treatment with DOP, the spleen index was increased, and this index may be related to the fatigue resistance.

## 4. Materials and Methods

### 4.1. Materials

A commercial *Rhodiola rosea* extract product containing 1% salidroside was used as the positive control. Triglyceride assay kits, lactic dehydrogenase assay kits, malonaldehyde assay kits, superoxide dismutase assay kits, glutathione peroxidase assay kits, lactic acid assay kits, urinary nitrogen assay kits, hepatic glycogen/muscle glycogen assay kits, creatine kinase assay kits were all purchased from Nanjing Jiancheng Bioengineering Institute (Nanjing, China). LPS (from *Escherichia coli* 0111:B4) and Concanavalin A (Con A) were purchased from Sigma-Aldrich (St. Louis, MO, USA). CellTiter 96^®^ AQueous One Solution Cell Proliferation kit was purchased from Promega Inc. (Madison, WI, USA). Polysaccharide marker DOP of *Dendrobium officinale* was prepared in our previous study [17].

### 4.2. Animals and Experimental Design

Inbred strain male (six to eight-week-old, 22 ± 2 g) BALB/c mice were purchased from the Laboratory Animal Services Centre of The Chinese University of Hong Kong. The animals were provided with standard pellet diet and water ad libitum and maintained under controlled conditions of temperature and humidity, with 12 h light/dark cycles. All experiments with animals were carried out in accordance with the Animals Ordinance, Department of Health, Hong Kong Special Administration Region, China for the care and use of experimental animals. All of the experimental protocols were first approved by the Committee on Use of Human and Animal Subjects in Teaching and Research of the Hong Kong Baptist University. The animals were used for weight-loaded swimming experiments after seven days of adaptation to the environment and the standard diet. Mice were trained to accustom themselves to swimming twice (10 min per time) in the first week. Mice which could not learn to swim were screened out. Trained mice were randomly divided into four groups, each consisting of eight mice.

Group 1 (Normal). Mice did not receive any treatment.

Group 2 (Control). Mice were given distilled water for 30 days, as a negative control.

Group 3 (DOP). Mice were treated with DOP (50 mg/kg) for 30 days.

Group 4 (PC). Mice were treated with *Rhodiola rosea* extract (100 mg/kg *) for 30 days, as a positive control.

* This dose corresponds to a typical human dose of 600 mg given to a 60 kg person (applying the coefficient equal 10 for adjusting differences between mouse and human in relation of the surface to body mass) according to the instruction of the *Rhodiola rosea* extract product.

DOP and *Rhodiola rosea* extract were dissolved in distilled water and fed by gavage to mice once a day. Changes in the body weight of the mice were observed every seven days. The above method of grouping and feeding was repeated to determine related indicators. Mice were anesthetized with chloral hydrate and blood samples were collected from each treatment group. Serum samples were obtained by centrifugation (3000 rpm, 10 min, 4 °C) and stored at −80 °C for further analysis. The spleens, hearts, and livers were weighed and their weights relative to the final body weights (organ index) were calculated.

### 4.3. Weight-Loaded Swimming Endurance Time

The procedure used in this assay followed a previous study described by Chen [35] and was similar to the methods described by Porsolt [36]. Briefly, 1 h after the last oral administration, mice were placed in the swimming pool (50 × 50 × 40 cm) filled with fresh water at 25 ± 1 °C, approximately 30 cm deep so that mice could not support themselves by touching the bottom with their feet. A tin wire (5% of body weight) was loaded on the tail root of the mouse. It was reported that this arrangement forced the mouse to maintain continuous rapid leg movement [37]. The swimming period was regarded as the time spent by the mouse floating in the water, struggling, until exhausted. The mice were assessed to be exhausted when they failed to rise to the surface of water to breathe within a 10 s period. At the end of the session, the mice were removed from the water, dried with paper towels, and placed back in their home cages.

### 4.4. Biochemical Analysis

After 28 days, the mice were taken out from each group for analyses of hepatic glycogen, muscle glycogen, and blood biochemical parameters. 1 h after the last intragastric administration of DOP and *Rhodiola rosea* extract, the mice were forced to swim in the swimming pool (weight-loaded) for a 6 min session according to the method in Section 4.3. At the end of the session, mice were removed from the water, dried with a paper towel, and anesthetized with an intraperitoneal injection of chloral hydrate. After anesthetization, blood was collected in heparinized tubes and tubes without anticoagulant by removing the left eyeball. Serum was prepared by centrifugation at 3500 rpm at 4 °C for 15 min. The blood plasma was tested to determine the concentration of lactic dehydrogenase (LDH), creatine phosphokinase (CK), triglyceride (TG), blood urea nitrogen (BUN), superoxide dismutase (SOD), malondialdehyde (MDA), lactic acid (LD), and glutathione peroxidase (GSH-Px) using commercial kits as listed in Section 4.1.

### 4.5. Analysis of Tissue Glycogen Contents

After the blood was collected, the livers and the gastrocnemius muscles of the mice were immediately dissected, frozen in liquid nitrogen, and kept at −80 °C until analysis of glycogen concentration. The concentration of hepatic glycogen was tested following the recommended procedures provided by the hepatic glycogen/muscle glycogen assay kits. Briefly, alkaline solutions were added to liver and gastrocnemius muscle samples for hydrolysis at 100 °C for 30 min. After centrifugation at 4000× *g* for 15 min, the supernatants were discarded. 0.5 mL of distilled water and 1 mL of 0.2% anthrone were added, and the vials were placed in a boiling-water bath for 20 min. The absorbance at 620 nm of the solutions in vials was determined by UV/VIS spectrophotometer (Jasco V530, Jasco, Tokyo, Japan).

### 4.6. Lymphocyte Proliferation Assays

Spleens were collected from BALB/c mice of each group after killing them by cervical dislocation. Single cell suspension of splenocytes was prepared according to the method described by Busse [38]. Briefly, the spleens were cut into several pieces and pressed through a 70 μm cell strainer (BD falcon, BD Biosciences, San Jose, CA, USA) into culture medium using a syringe plunger. Spleen cells were re-suspended in red cell lysis buffer and incubated at room temperature for 5 min. The resulting pellet was re-suspended and diluted to 5 × 10^6^ cells/mL with RPMI-1640 after the cell viability was assessed by trypan blue exclusion. The 100 μL cell suspension was incubated in 96-well culture plates. It is known that LPS and Con A stimulate B cells and T cells, respectively [39]. Thus, the lymphocyte proliferation was tested by incubating the mouse lymphocytes in the absence or presence of LPS and Con A at the optimal concentration (LPS: 20 μg/mL and Con A: 2.5 μg/mL) for 48 h. After that, 20 μL of CellTiter 96^®^ AQueous One Solution Cell Proliferation reagent was added into each well at 4 h before the end of incubation. The absorbance of cells in each well was measured by Benchmark Plus microplate reader (Bio-Rad, Richmond, CA, USA) at a wavelength of 490 nm.

### 4.7. Statistical Analysis

All values are expressed as means ± standard error (S.E.) in the tables and are indicated by vertical bars in the figures. Data were analyzed by one-way ANOVA, and then differences among means were analyzed using Fisher’s protected least significant differences (LSD) multi-comparison test. Differences were considered significant at *p* < 0.05.

## 5. Conclusions

In this paper, unique polysaccharide marker of *Dendrobium Officinale*, named DOP, have been used to study the anti-fatigue effect on BALB/c mice. Using the weight-loaded swimming test, DOP shows stronger anti-fatigue activity than that of positive control (*Rhodiola rosea* extract). In addition, the several serum parameters, indicating the fatigue symptom, were blocked to a certain extent by both DOP and Rhodiola rosea extract, compared with those of the control group. Furthermore, DOP-feeding mice significantly increased the cell variability of T lymphocytes and B lymphocytes, compared with that of mice in control group. These results indicate that the anti-fatigue effects of DOP due to the enhanced triglyceride (TG) (or fat) mobilization, decreased lipid oxidation and elevated immunomodulating activity during exercise in mice. Although our work is limited to the mechanism study, we can expect that DOP has the promising potential for pharmaceutical development into health products to reduce fatigue.

## Supplementary Materials

Supplementary materials can be accessed at: http://www.mdpi.com/1420-3049/22/1/155/s1.

## Abbreviations

DOP	*Dendrobium officinale* polysaccharide
LDH	Lactic dehydrogenase
CK	Creatine phosphokinase
TG	Triglyceride
BUN	Blood urea nitrogen
SOD	Superoxide dismutase
MDA	Malondialdehyde
LD	Lactic acid
GSH-Px	Glutathione peroxidase
AIDS	Acquired Immune Deficiency Syndrome
LPS	Lipopolysaccharide
Con A	Concanavalin A
ROS	Reactive oxygen species

## References

[1] Chaudhuri A., Behan P.O. (2004). Fatigue in neurological disorders. Lancet.

[2] Kroenke K., Wood D.R., Mangelsdorff A.D., Meier N.J., Powell J.B. (1988). Chronic fatigue in primary care: Prevalence, patient characteristics, and outcome. JAMA.

[3] Papanicolaou D.A., Amsterdam J.D., Levine S., McCann S.M., Moore R.C., Newbrand C.H., Allen G., Nisenbaum R., Pfaff D.W., Tsokos G.C. (2004). Neuroendocrine aspects of chronic fatigue syndrome. Neuroimmunomodulation.

[4] Li M., Donglian C., Huaixing L., Bende T., Lihua S., Ying W. (2008). Anti-fatigue effects of salidroside in mice. J. Med. Coll. PLA.

[5] Darbinyan V., Kteyan A., Panossian A., Gabrielian E., Wikman G., Wagner H. (2000). *Rhodiola rosea* in stress induced fatigue—A double blind cross-over study of a standardized extract SHR-5 with a repeated low-dose regimen on the mental performance of healthy physicians during night duty. Phytomedicine.

[6] Levine P.H., Whiteside T.L., Friberg D., Bryant J., Colclough G., Herberman R.B. (1998). Dysfunction of natural killer activity in a family with chronic fatigue syndrome. Clin. Immunol. Immunopathol..

[7] Ojo-Amaize E.A., Conley E.J., Peter J.B. (1994). Decreased natural killer cell activity is associated with severity of chronic fatigue immune dysfunction syndrome. Clin. Infect. Dis..

[8] Buchwald D., Wener M., Pearlman T., Kith P. (1997). Markers of inflammation and immune activation in chronic fatigue and chronic fatigue syndrome. J. Rheumatol..

[9] Landay A., Lennette E., Jessop C., Levy J. (1991). Chronic fatigue syndrome: Clinical condition associated with immune activation. Lancet.

[10] Mawle A.C., Nisenbaum R., Dobbins J.G., Gary H.E., Stewart J.A., Reyes M., Steele L., Schmid D.S., Reeves W.C. (1997). Immune responses associated with chronic fatigue syndrome: A case-control study. J. Infect. Dis..

[11] Ng T.B., Liu J., Wong J.H., Ye X., Sze S.C.W., Tong Y., Zhang K.Y. (2012). Review of research on dendrobium, a prized folk medicine. Appl. Microbiol. Biotechnol..

[12] Yi F., Huang M.K., Ye J.B., Ning L., Zhong W.G. (2014). The lowest doses of *Dendrobium officinale* for improving sports ability, anti-fatigue and immune ability of mice. J. South. Agric..

[13] Xu J., Li S.-L., Yue R.-Q., Ko C.-H., Hu J.-M., Liu J., Ho H.-M., Yi T., Zhao Z.-Z., Zhou J. (2014). A novel and rapid hpgpc-based strategy for quality control of saccharide-dominant herbal materials: Dendrobium officinale, a case study. Anal. Bioanal. Chem..

[14] Cai H.-L., Huang X.-J., Nie S.-P., Xie M.-Y., Phillips G.O., Cui S.W. (2015). Study on *Dendrobium officinale* o-acetyl-glucomannan (dendronan^®^): Part III—Immunomodulatory activity in vitro. Bioact. Carbohydr. Diet. Fibre.

[15] Liu X.-F., Zhu J., Ge S.-Y., Xia L.-J., Yang H.-Y., Qian Y.-T., Ren F.-Z. (2011). Orally administered *Dendrobium officinale* and its polysaccharides enhance immune functions in balb/c mice. Nat. Prod. Commun..

[16] Xia L., Liu X., Guo H., Zhang H., Zhu J., Ren F. (2012). Partial characterization and immunomodulatory activity of polysaccharides from the stem of *Dendrobium officinale* (tiepishihu) in vitro. J. Funct. Foods.

[17] Wei W., Feng L., Bao W.-R., Ma D.-L., Leung C.-H., Nie S.-P., Han Q.-B. (2016). Structure characterization and immunomodulating effects of polysaccharides isolated from dendrobium officinale. J. Agric. Food Chem..

[18] Dubovik B.V., Bogomazov S.D. (1987). Multifactorial method for assessing the physical work capacity of mice. Farmakol. Toksikol..

[19] Öztürk N., Başer K.H.C., Aydin S., Öztürk Y., Çaliş I. (2002). Effects of gentiana lutea ssp. Symphyandra on the central nervous system in mice. Phytother. Res..

[20] Yu N.X. (2005). Method for Preparing Compound Micro *Dendrobium officinale* Kimura et Migo Granule and Capsule for Nourishing, Reducing Blood Sugar and Anti-Fatigue. Chinese Patents.

[21] Langdon W.B. (2015). Performance of genetic programming optimised bowtie2 on genome comparison and analytic testing (GCAT) benchmarks. BioData Min..

[22] Lv G., Yan M., Chen S. (2013). Review of pharmacological activities of *Dendrobium officinale* based on traditional functions. China J. Chin. Mater. Med..

[23] Wakayoshi K., Yoshida T., Udo M., Kasai T., Moritani T., Mutoh Y., Miyashita M. (1992). A simple method for determining critical speed as swimming fatigue threshold in competitive swimming. Int. J. Sports Med..

[24] Kim K., Yu K., Kang D., Suh H. (2002). Anti-stress and anti-fatigue effect of fermented rice bran. Phytother. Res..

[25] Selsby J.T., Beckett K.D., Kern M., Devor S.T. (2003). Swim performance following creatine supplementation in division iii athletes. J. Strength Cond. Res..

[26] Hauger R.L., Millan M.A., Lorang M., Harwood J.P., Aguilera G. (1988). Corticotropin-releasing factor receptors and pituitary adrenal responses during immobilization stress. Endocrinology.

[27] Wójcik R., Siwicki A., Skopinska-Rózewska E., Wasiutynski A., Sommer E., Furmanowa M. (2009). The effect of chinese medicinal herb rhodiola kirilowii extracts on cellular immunity in mice and rats. Pol. J. Vet. Sci..

[28] Sommer E., Mielcarek S., Buchwald W., Krajewska-Patan A. (2007). The influence of *Rhodiola rosea* extracts on non-specific and specific cellular immunity in pigs, rats and mice. Cent. Eur. J. Immunol..

[29] Guan S., He J., Guo W., Wei J., Lu J., Deng X. (2011). Adjuvant effects of salidroside from *Rhodiola rosea* L. on the immune responses to ovalbumin in mice. Immunopharmacol. Immunotoxicol..

[30] Skopińska-Różewska E., Bychawska M., Białas-Chromiec B., Sommer E. (2009). The in vivo effect of *Rhodiola rosea* and rhodiola quadrifida hydro-alcoholic extracts on chemokinetic activity of spleen lymphocytes in mice. Cent. Eur. J. Immunol..

[31] Dorchy H. (2002). Sports and type I diabetes: Personal experience. Rev. Med. Brux..

[32] Singh A., Naidu P.S., Gupta S., Kulkarni S.K. (2002). Effect of natural and synthetic antioxidants in a mouse model of chronic fatigue syndrome. J. Med. Food.

[33] Lorusso L., Mikhaylova S.V., Capelli E., Ferrari D., Ngonga G.K., Ricevuti G. (2009). Immunological aspects of chronic fatigue syndrome. Autoimmun. Rev..

[34] Huang X., Nie S., Cai H., Zhang G., Cui S.W., Xie M., Phillips G.O. (2015). Study on *Dendrobium officinale O*-acetyl-glucomannan (dendronan): Part iv. Immunomodulatory activity in vivo. J. Funct. Foods.

[35] Chen Y., Kong L.D., Xia X., Kung H.F., Zhang L. (2005). Behavioral and biochemical studies of total furocoumarins from seeds of psoralea corylifolia in the forced swimming test in mice. J. Ethnopharmacol..

[36] Porsolt R., Bertin A., Jalfre M. (1977). Behavioral despair in mice: A primary screening test for antidepressants. Arch. Int. Pharmacodyn. Ther..

[37] Boström S., Fahlen M., Hjalmarson A., Johansson R. (1974). Activities of rat muscle enzymes after acute exercise. Acta Physiol. Scand..

[38] Busse C.E., Czogiel I., Braun P., Arndt P.F., Wardemann H. (2014). Single-cell based high-throughput sequencing of full-length immunoglobulin heavy and light chain genes. Eur. J. Immunol..

[39] Lin K.-H., Lin K.-C., Lu W.-J., Thomas P.-A., Jayakumar T., Sheu J.-R. (2015). Astaxanthin, a carotenoid, stimulates immune responses by enhancing ifn-γ and il-2 secretion in primary cultured lymphocytes in vitro and ex vivo. Int. J. Mol. Sci..

