# Supplementary material for: Anti-Fatigue Effects of the Unique Polysaccharide Marker of Dendrobium officinale on BALB/c Mice
Source: Molecules. 2017 Jan 18;22(1):155. doi: 10.3390/molecules22010155 (PMC6155575; doi:10.3390/molecules22010155)
Supplement: Supplementary file 1 [file molecules-22-00155-s001.pdf]

Supplementary Materials: Anti-Fatigue Effects of the Unique Polysaccharide Marker of *Dendrobium Officinale* on BALB/c Mice

Wei Wei, Zhi-Peng Li, Tong Zhu, Hau-Yee Fung, Tin-Long Wong, Xin Wen, Dik-Lung Ma, Chung-Hang Leung and Quan-Bin Han

Table S1. Effects of DOP and *Rhodiola* extract on body weight (g) of BALB/c mice.

	Day 1	Day 7	Day 14	Day 21	Day 28
Normal group	21.54 ± 0.52	22.18 ± 0.52	22.74 ± 0.72	23.28 ± 1.05	24.08 ± 0.77
Control group	22.53 ± 0.43	22.54 ± 0.63	22.70 ± 0.98	23.68 ± 0.77	23.96 ± 0.91
DOP group	22.42 ± 1.02	22.84 ± 0.93	23.16 ± 1.29	24.10 ± 1.43	24.59 ± 1.54
PC group	23.38 ± 0.33	23.21 ± 0.72	23.61 ± 0.87	24.44 ± 1.17	24.76 ± 0.38

The data were presented as the mean ± SD (n = 8). Normal group means mice were unexposed to weight-loaded swimming endurance test. Control group means mice were exposed to weight-loaded swimming endurance test and treated with distilled water. PC group means positive group, *Rhodiola* extract group.

Table S2. Effects of DOP and *Rhodiola* extract on food intake (g) of BALB/c mice.

	First Week	Second Week	Third Week	Fourth Week
Normal group	186.3	182.1	185.4	188.1
Control group	194.8	185.4	194.0	197.5
DOP group	203.3	214.8	221.2	222.7
PC group	202.6	197.5	200.3	206.7

Normal group means mice were unexposed to weight-loaded swimming endurance test. Control group means mice were exposed to weight-loaded swimming endurance test and treated with distilled water. PC group means positive group, *Rhodiola* extract group.

Table S3. Effects of DOP and *Rhodiola* extract on organ index of BALB/c mice.

	Liver	Heart	Kidney	Spleen
Normal group	53.70 ± 1.90	6.43 ± 0.85	15.84 ± 0.73	4.37 ± 0.32
Control group	52.73 ± 1.30	6.36 ± 0.73	15.75 ± 0.43	4.17 ± 0.47
DOP group	54.25 ± 2.52	6.86 ± 0.27	16.60 ± 1.18	4.86 ± 0.49
PC group	53.44 ± 2.62	6.44 ± 0.70	15.60 ± 1.17	5.34 ± 0.68

The data were presented as means ± S.D. (n = 8). Organ index = weight of organ (mg)/body weight (g). PC group means positive group, *Rhodiola* extract group.
